# Conduction system pacing: promoting the physiology to prevent heart failure

**DOI:** 10.1007/s10741-023-10296-4

**Published:** 2023-02-14

**Authors:** Han Naung Tun, Hafiza Khan, Daryna Chernikova, Yury Mareev, Santabhanu Chakrabarti, May Thant, Antonio Cannata

**Affiliations:** 1 UVM Medical Centre, Larner College of Medicine, University of Vermont, Given Medical Bldg, E-126, 89 Beaumont Ave, Burlington, VT 05405 USA; 2 Cardiac Electrophysiology, Baylor Scott & White The Heart Hospital, TX Plano, USA; 3 Cardiology Department, City Hospital, Heroiv Ukrainy, 17 Street, 84300 Kramatorsk Donetsk, Ukraine; 4 Department of Cardiology, National Medical Research Centre for Therapy and Preventive Medicine, Moscow, Russia; 5 Robertson Centre for Biostatistics, University of Glasgow, Glasgow, UK; 6 Division of Cardiology, Department of Medicine, University of British Columbia, Heart Rhythm Services, 211-1033 Davie Street, Vancouver, BC V4N 0J9, Canada; 7 Royal Blackburn Hospital, Health Education England, Northwestern Deanery, Haslingden Rd, Blackburn, BB2 3HH UK; 8 Department of Cardiovascular Sciences, Faculty of Life Sciences & Medicine, King’s College - London, London, UK

**Keywords:** His bundle pacing, Left bundle area pacing, Cardiac resynchronization, Heart failure

## Abstract

Cardiac conduction system pacing provides physiological ventricular activation by directly stimulating the conduction system. This review describes the two types of conduction system pacing: His bundle pacing (HBP) and left bundle area pacing (LBAP). The most significant advantage of HB pacing is that it can provide a regular, narrow QRS; however, the disadvantages are challenging implantation and a high risk of re-intervention due to lead dislodgement and the development of high pacing threshold. LBAP provides optimum physiological activation of the left ventricle by engaging the left bundle/fascicular fibers. LBAP is more physiological than traditional RV apical pacing and could be an attractive alternative to conventional cardiac resynchronization therapy (CRT). The advantages of LBAP are a relatively more straightforward implantation technique than HBP, better lead stability and pacing thresholds. HBP and LBAP are more physiological than right ventricular pacing and may be used instead of conventional pacemakers. Both HBP and LBBP are being investigated as alternatives to conventional CRT.

## Introduction

Conventional cardiac resynchronization therapy (CRT) is an essential part of treating selected patients with HFrEF and ventricular dyssynchrony due to wide QRS. However, conventional CRT requires implantation of an extra lead in the coronary sinus, which sometimes may be challenging and less feasible due to unsuitable anatomy, phrenic nerve stimulation, and unacceptably high local thresholds. Moreover, the left ventricular epicardial stimulation may not entirely resolve the electrical dyssynchrony [1, 2]. Epicardial placement of LV lead has been suggested as an alternative method in the case of transvenous procedure failure during CRT device implantation, but it may cause lead failure and rather highly reported complications such as infection [5].

These challenges with conventional CRT led to the development of a new concept of pacing—the conduction system pacing (namely His bundle pacing (HBP) and left bundle area pacing (LBAP)), which can also be alternatives to conventional right ventricular pacing in selected patients.

## Clinical anatomy of physiological pacing

The His bundle (HB) is a thin structure penetrating the central fibrous body of the heart and has two main anatomical variants. The type I HB, present in 46.7% of subjects, when the AV bundle is covered by a thin layer of myocardial fibers and runs along the lower border of the membranous septum. Conversely, the type II HB runs within the muscular part of the interventricular septum below the pars membranacea [2, 3]. Both atrial and ventricular portions of the HB can be accessed for permanent conduction system pacing [1]. The HB has significant positional variations relative to the membranous septum [1], influencing selective His bundle pacing (S-HBP) or nonselective His bundle pacing (NS-HBP) during permanent HBP procedure.

LBB’s anatomical features determine the feasibility of LBBP as a potential physiological pacing modality. In contrast to HBP, LBBP that is determined by the capture of the LBB and distal conduction system tissues has a much wider target zone for area pacing that is likely to be beyond the site of the block in distal HB [5]. In contrast to right ventricular apical pacing, HBP does not induce interventricular or intraventricular asynchrony and does not provoke myocardial perfusion disorders [6].

## Development and early experience of human HBP and LBBP

Implementing physiological pacing techniques directly activating the conduction system has been and continues to be a crucial issue in managing cardiac conduction disease. Hence, electrophysiological challenges arose as development progressed. Nearly 5 decades ago Narula et al. reported that the pacing impulse to ventricular activation time (PI-R) during the procedure was the same as the H-V time during normal sinus rhythm [7]. Subsequently, the ability of HBP to generate truly physiological ventricular activation would allow this technique to become a full-fledged alternative to cardiac resynchronization therapy (CRT) [8] and has been recommended as a rescue modality for failed biventricular pacing [9].

Since 2006, several reports which described the use of HBP in clinical practice have been published [10]. These reports have led to further investigation of the effectiveness of permanent HBP in patients requiring pacing and device-paced HF therapy [11]. After that, the benefits of permanent HBP were proved in multiple studies. In particular, the first systematic analysis of a large pool of patients demonstrated a high success for HBP concerning the sustaining of cardiac function with the potential for significant improvement in LVEF in patients with systolic dysfunction and heart failure [12].

**Fig. 1:**
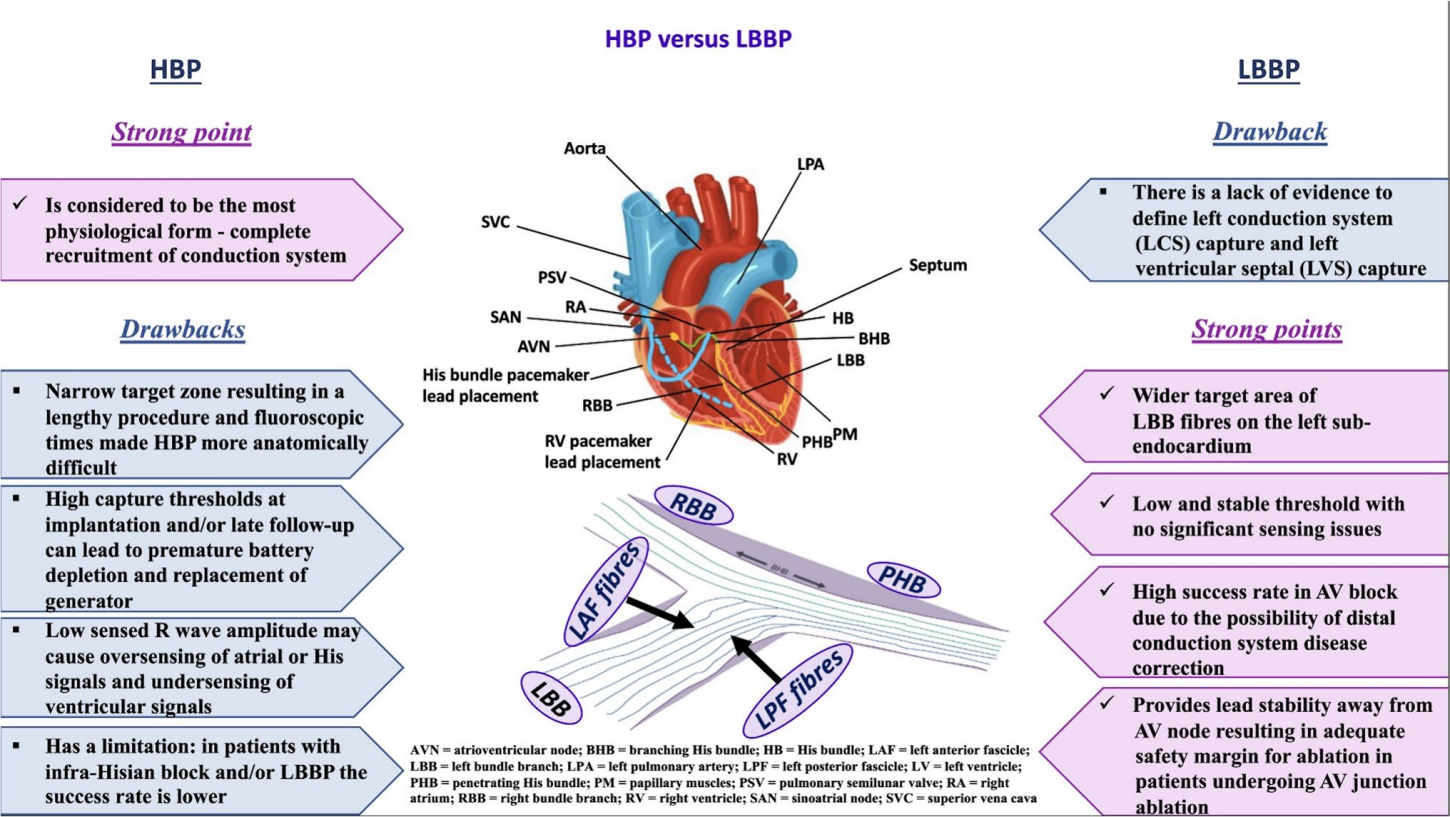
Advantages and disadvantage of His bundle pacing (HBP) vs left bundle branch pacing (LBBP)

However, due to significant procedural limitations and technical complexities of HBP associated with the risk of causing distal conduction block, high capture threshold, and low sensed R wave amplitude (Fig. 1), researchers and clinicians have faced the necessity to develop a better pacing modality for delivering physiological pacing, the LBAP therapy [13]. After that, the advantages of the LBAP technique in patients with cardiomyopathy have been demonstrated across several studies [14].

## Mechanisms for LBBB reversal with His bundle pacing

His bundle pacing (HBP) has arisen as a novel and alternative method for cardiac resynchronization therapy (CRT) in patients with heart failure (HF) and left bundle branch block (LBBB). The main reason for implementing effective ventricular resynchronization and more physiological activation is the obvious improvement in cardiac function. This method can also significantly reduce QRS duration and restore normal intrinsic activation patterns in patients with ventricular conduction delays, as demonstrated by Ali et al. [21].

Thus, for the first time, the concept of longitudinal dissociation with the fibers within the His bundle committed to the left bundle with asynchronous conduction resulting in an LBBB pattern was described. The study showed that localized lesions within the His bundle induced LBBB. Meanwhile, stimulation of the HB proximal to the intra-His lesion could lead to ventricular depolarization. HB stimulation at a site distal to the lesion, in turn, resulted in narrow QRS complexes due to synchronous impulse conduction to LBB and RBB. These fundamental concepts confirmed the feasibility of HBP, including in advanced His to ventricular electrogram interval (HV) disease stages [6].

In some cases, the factors like the higher lead revision rate or pacing thresholds can prevent HBP from reversing LBB. Concerning the mechanisms for HBP narrowing or reversing BBB, there are the following:

- The pacing lead is placed distal to the site of BBB fibers within the HB are ordered in strands predestined for the LBB or RBB. The position of a pacing lead can reverse conduction delays within the HB [25].
- The connection between source and sink: the block is overcome with sufficient stimulus for activation of distal dormant tissue based on the source-sink connection during pacing versus intrinsic impulse propagation [25].
- Retrograde activation: the activation of the His-Purkinje system happens through the capture of an upper septal branch that permits onward antegrade activation beyond a block site [25].

## Acute and chronic effects of HBP and LBBP

In a prospective crossover study, Catanzariti et al. proved that during direct HBP, physiologic distribution of myocardial blood flow was preserved more in comparison with right ventricular apical pacing [26]. In another crossover trial, the evaluation of myocardial perfusion and mitral regurgitation showed significant improvement in the assessed indicators. Still, HBP, in this case, had no effect on LV systolic function [27]. The results of the study by Zanon et al., meanwhile, demonstrated that HBP mode in comparison with RV apical pacing in patients undergoing permanent implantation of a HBP lead contributed to improvement in LV systolic function and echocardiographic indices of ventricular synchrony [12].

In a more recent study, where the effects of HBP in patients with LV systolic dysfunction, first-degree atrioventricular block (AVB), and either RBBB or narrow QRS complex were evaluated, it was proved that temporary HBP did not lead to an increase in QRS duration (in comparison with temporary biventricular pacing) [28]. A randomized study by Ellenbogen and Huizar demonstrated that LVEF was significantly higher after 12 months of His pacing in patients compared with AVB, narrow QRS, and LVEF > 0.40 as compared with RVP [29]. Evaluating the long-term lead performance of His pacing, Chen et al. reported successful HBP in 80% of cases with markedly lower death or HF hospitalization in HBP compared to RVP patients at five years of follow-up [30].

## HBP and LBBP in CRT-eligible populations

Several prospective randomized studies proved the effects of BVP in reducing mortality rates and heart failure hospitalization, improving quality of life, and increased exercise capacity. However, the CRT non-response is up to 30% in all CRT candidates [31]. In patients with narrow QRS or moderate QRS prolongation (i.e., < 130 ms), BVP can cause prolongation of ventricular activation time and worsen dyssynchronous activation [32]. Furthermore, right ventricular pacing should not be applied to patients with impaired LVEF to avoid the development of pacing-induced cardiomyopathy. Since BVP does not deliver true physiological pacing, it has not demonstrated superiority over RVP in patients with LVEF > 45%, according to the results of the BIOPACE trial [33].

A multicenter study by Ali et al. evaluated the feasibility and outcomes of the LBBAP method in CRT-eligible patients or those who underwent unsuccessful CRT. In this cohort study, all patients had NYHA class II to IV, baseline LVEF ≤ 50%, and indications for ventricular pacing and/or CRT. Based on the results, LBBAP was associated with reduced paced QRS duration, improving clinical and echocardiographic outcomes. Hence, LBBAP can be a feasible, safe, and potentially an alternative option for CRT [21]; however, this option needs to be tested in large clinical trials.

In a randomized crossover study, Gasparini et al. demonstrated no significant difference in clinical and echocardiographic improvements while applying HBP compared with BVP [34]. However, certain disadvantages of HBP, such as higher pacing thresholds and the inability to correct distal LBBB, were a limitation for using this technique, as demonstrated by Leclercq et al. comparing HBP with BVP [35].

## Clinical perspectives of His bundle pacing and left bundle branch pacing area in heart failure

Cardiac resynchronization therapy is the gold standard in the management of patients with systolic heart failure and electromechanical dyssynchrony, as evidenced by a wide QRS duration. For over two decades, the resynchronization method has been through by ventricular pacing. We have strong data using prospective randomized studies that show biventricular pacing improves quality of life New York Heart Association classification, left ventricular ejection fraction, and left ventricular volumes. However, despite strong clinical evidence regarding the efficacy of biventricular pacing, it is estimated that approximately 1/3 of patients have no clinical benefit or response to CRT via LV lead placement (Table 1).

**Table 1:** Current limitations of HBP and LBBP in heart failure

**HBP**	**LBBAP**
1. Higher pacing thresholds 2. Increased implantation time and fluoroscopy time 3. Lead dislodgement 4. Elevated pacing thresholds at follow-up 5. Rapid battery depletion due to an increase in pacing thresholds 6. Failure to achieve His bundle capture 7. Absence of a unique CPT code resulting in no increase in payment despite increased physician time utilization, EP lab utilization, and use of additional sheaths and equipment 8. Lack of large RCT data regarding outcomes in comparison to traditional CRT 9. Nuanced implant technique, limited to mostly electrophysiologists 10. Not scalable 11. High reintervention rate 12. Concerns regarding aortic valve endocarditis if lead related infection	1. Learning curve regarding the use of the delivery systems of several vendors 2. Learning curve regarding the left bundle branch area pacing site and placement of the lead deep into septal 3. Risk of deep septal lead placement, including but not limited to perforation into the left ventricle 4. Potential for higher complication rate should the deep intra-septal lead need to be extracted in the future 5. Absence of big randomized controlled data comparing left bundle branch area pacing in patients requiring CRT in comparison to traditional biventricular pacing 6. Accurate understanding of criteria for left bundle branch area capture by implanting physician 7. Long-term (> 12 months) lead performance with deep septal implantation of pacing lead 8. Increased procedure time and fluoroscopy time, in the initial implant learning curve 9. No unique CPT code; hence, no payment for increased physician procedure time and EP lab time utilization 10. Equipment issues: new lead design and delivery systems are being designed

### HBP

**Fig. 2:**
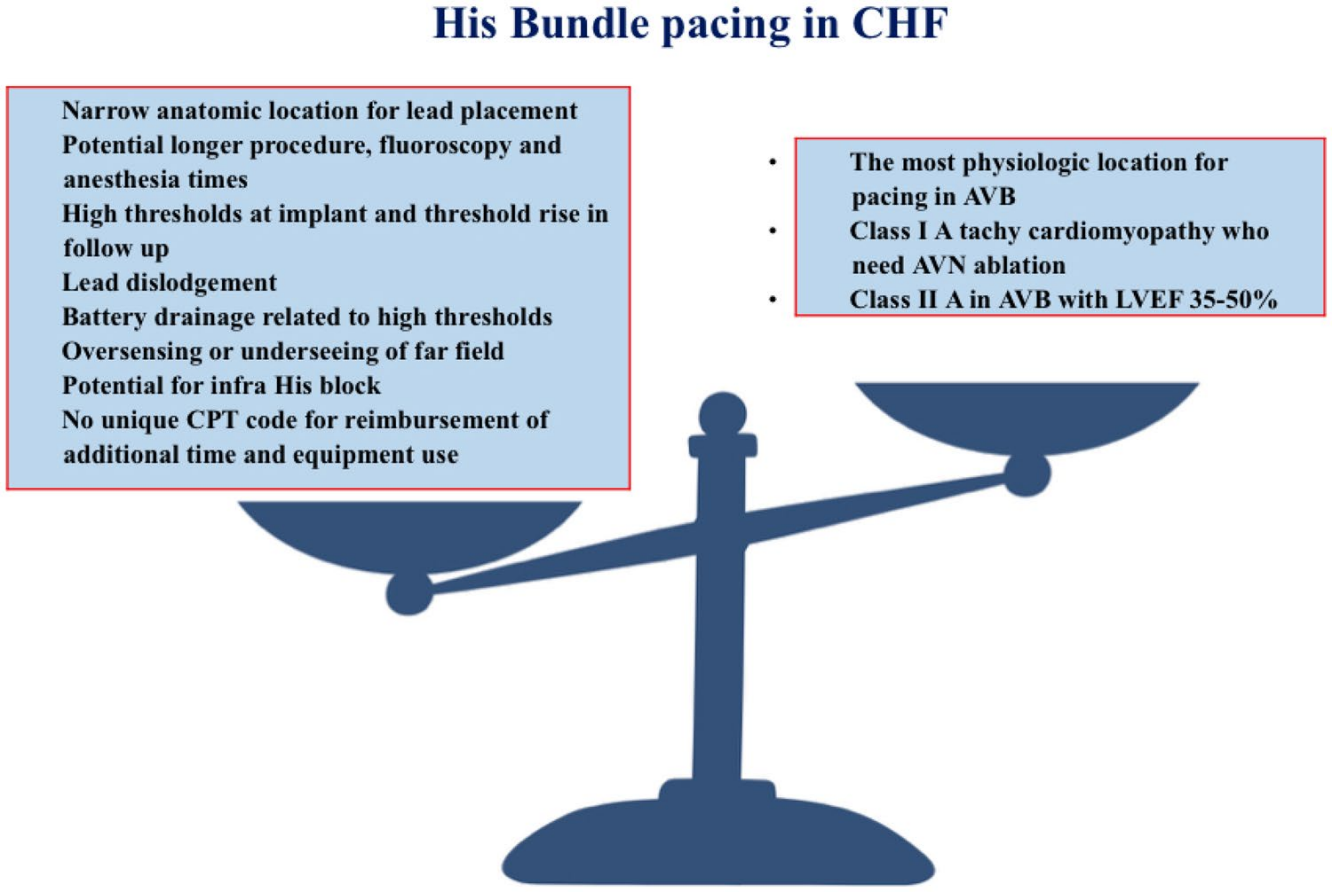
His bundle pacing in CHF

Conduction system pacing, either via His bundle pacing or more recently left bundle branch pacing, has emerged as a viable alternative to traditional left ventricular CRT in patients with congestive heart failure. His bundle pacing via activation of the His-Purkinje conduction system and resultant physiologic ventricular activation has been promoted as a favorable alternative to Bi ventricular pacing strategy in patients with and without heart failure (Fig. 2).

### LBBAP

Left bundle branch area pacing has been suggested as an effective alternative to overcome the limitations of His bundle pacing. Left bundle branch pacing allows for physiologic stimulation of the left bundle component of cardiac conduction system like HBP, however, with improved leads to stability and fewer implant and post-op technologic challenges. Left bundle branch pacing has a larger target area and somewhat fewer technological implant challenges. Lead stability and thresholds also appear to be improved compared to His bundle pacing. Nonetheless, it is important to understand anatomy and fluoroscopic views when implantation of the lead to achieve accurate left bundle capture.

Vijayaraman et al. recently published their data regarding the feasibility and outcomes of left bundle branch area pacing for CRT in a multicenter international collaborative study. LBBP was attempted in 325 patients with LVEF < 50% and an indication for CRT. CRT was successfully achieved in 277 of these patients (85%). QRS configuration at baseline was left bundle branch block in 39%, and non-left bundle branch block in 46%. Procedure times were 105 ± 54 min, and fluoroscopy time was acceptable at 19 ± 15 min. Importantly, left bundle branch area pacing thresholds were 0.6 ± 0.3 V @0.5 ms, and R wave amplitude was acceptable at 10.6 ± 6 mV at implantation. Sensing and thresholds remain stable during the follow-up of approximately 6 months. The importantly clinical and echocardiographic response was observed in 72 to 73% of the patients who achieved left bundle branch pacing. This study proved that left bundle branch pacing is a feasible alternative for cardiac resynchronization therapy providing acceptable pacing and sensing parameters both in the short and long term with no excessive procedure times and successful clinical outcomes.

Recent randomized LBBP-RESYNC study (40 pts) showed that patient randomized to LBBP has higher LVEF improvement. In this study, 10% LBBP patients were crossover to biventricular CRT and 20% of biventricular CRT were crossover to LBBB pacing due to the problems with lead placement. Which on the one hand showed that is not always possible to perform both of techniques in one patient but in case of the impossibility of one of the methods it is possible to change to another. Both HBP and LBAP could be helpful also in patients without HF in whom we expect a high rate of RV stimulation. HBP is the most physiological pacing modality that restores normal ventricular activation and has been demonstrated to achieve greater hemodynamic response over BVP in patients [44]. Confirmation of LBB capture is essential to distinguish LBBP from LVSP, as LBBP ensures rapid LV activation propagation via conduction system rather than myocardial endocardium and hence improves ventricular electrical synchrony.

## Pacing strategies for HF and AF patients

Atrial fibrillation (AF), the most common arrhythmia, increases the risk of death and hospitalization in 76% of HF patients, and the structural and neurohormonal changes in HF make, in turn, the development and progression of AF much more likely [15]. AF ablation was associated with a significant improvement in LVEF, independent of the severity of left ventricular dysfunction [16].

In a study, Molhoek et al. patients with AF showed a milder degree of response to CRT compared to those with sinus rhythm. However, the long-term survival rate was comparable among these two groups of patients [36, 37]. According to the guidelines, CRT should be performed in patients with HF and LVEF ≤ 35% with NYHA class III or IV if they are in AF and have intrinsic QRS ≥ 130 ms, provided a strategy to ensure biventricular capture is in place. Meanwhile, AV junction ablation should be added in the case of incomplete biventricular pacing (< 90–95%) due to conducted AF [38].

An intrinsic and irregular spontaneous AF rhythm reduces the percentage of effectively biventricular paced captured beats, making CRT delivery more challenging in those patients with AF [39]. Nevertheless, the deleterious hemodynamic effects of irregular, spontaneous rhythm could be eliminated by AVJ ablation delivery. Hence, in the context of CRT in patients with HF and concomitant AF, many studies have shown AVJ ablation’s benefits for optimization of CRT procedure [40]. The MUSTIC AF trial is considered the first randomized trial showing potential benefits of CRT in HF patients with permanent AF by determining biventricular stimulation as a preferred mode compared to RV [41]. An observational study by Gasparini et al. demonstrated that significant improvements in LVEF, the left ventricular end-systolic volume (LVESV), and exercise capacity were observed in AF patients who underwent AVJ ablation [37, 38]. Deshmukh et al. demonstrated further improvement in LV dimensions with His pacing in patients with impaired LV systolic function and AF prior to AV node ablation and achieved procedural success in 60% of cases [36, 37].

On the other side, in selected patients with AF and HF, especially with uncontrolled heart rate, the “ablate-and-pace” strategy can be beneficial, resulting in improvement of LVEF and the NYHA functional class [17]. However, potential downsides of such a strategy are the risk of progressive left ventricular dyssynchrony, deterioration of LVEF, and the risk of sudden death after AV node ablation [18]. To prevent mechanical ventricular dyssynchrony and further HF aggravation, cardiac resynchronization therapy (CRT) is an effective option, although patients with AF show a milder degree of improvement with CRT compared with patients with sinus rhythm [19].

In patients with HF and sinus rhythm, PR prolongation is a prospective issue for pacing. A PR ≥ 200 ms is significantly associated with 58% higher mortality in the long term regardless of QRS duration [20]. According to the results of the studies, the prevalence of prolonged PR in patients with HF and CRT stands at 18–52% [21].

The benefits of HBP in HF can potentially apply to patients with narrow QRS and PR prolongation by providing AV synchrony without inducing ventricular dyssynchrony [22]. The EuroHeart Failure survey identified that about 75% of patients hospitalized with a suspected diagnosis of HF had normal QRS duration (≤ 120 ms) [23]. Meanwhile, up to 50% of HF patients with a narrow QRS complex show echocardiographic evidence of ventricular dyssynchrony and hence might benefit from CRT, resulting in frequent off-label use of CRT [24]. Noteworthy, HBP has a higher success rate in patients with symptomatic AV block if the level is nodal compared to infranodal [4]. Anatomy of the mechanism of pacing procedures should be considered during maneuvers to ensure that the lead is positioned distal to the site of diseased HB [4].

At present with the development of dedicated tools for direct HBP, the success rate of implantations has become more than 90%. Moreover, in most cases, the acceptable pacing thresholds can be achieved [37]. Su et al. evaluated the long-term performance of HBP following AV node ablation in patients with AF and HF. It was demonstrated that HBP combined with AV node ablation was effective in AF patients with drug-refectory HF. Furthermore, high pulmonary artery systolic pressure (PASP), elevated serum creatinine (Scr), and low LVEF at baseline were established as independent predictors of the composite endpoint of all-cause mortality or HF hospitalization [38].

The study by Vinther et al. observed HF patients with LBBB, demonstrated that His-CRT provided similar clinical improvement in comparison with BiV-CRT at the expense of higher pacing thresholds [39]. One more study successfully achieved permanent HBP in 80% of patients with AF, with narrow QRS duration, both HF with a preserved ejection fraction (HFpEF) and HF with reduced ejection fraction (HFrEF). The results of the study demonstrated a reduction in hospital admissions as well as an improvement in cardiac function [40].

## Physiological pacing in patients with heart failure with preserved ejection fraction

Increase heart rate (HR) may have the potential to reduce the risk for heart failure hospitalization, atrial fibrillation (AF), and cerebrovascular stroke as these outcomes are increased in patients with a normal or preserved ejection fraction on HR-lowering treatments. Therefore, lower HR elevation employing physiological conduction system pacing in patients with HFpEF will decrease left atrial and left ventricular filling pressures. There is an ongoing randomized trial that is investigating whether pacing with a higher heart rate is beneficial for patients with HFpEF and pacemakers with intrinsic AV conduction or CRT. Also, physiological pacing (CRT or His) could be beneficial in patients with HF, LVEF 35–50%, and indications for pacemaker implantation with high expected percent of pacing (BLOCK HF study) [42]. Again, physiologic accelerated pacing as a treatment in patients with heart failure with preserved ejection fraction (PACE HFpEF) trial is now under investigation hypothesizes that a personalized lower HR elevation employing physiological conduction system pacing in patients with HFpEF will decrease left atrial and left ventricular filling pressure [43].

## Conclusion

Implementing physiological pacing techniques directly activating the conduction system has been and continues to be a crucial issue in managing cardiac conduction disease. HBP is the most physiological pacing modality that restores normal ventricular activation and has been demonstrated to achieve greater hemodynamic response over BVP in patients. HBP combined with AV node ablation showed effectiveness in AF patients with drug-refectory HF. Left bundle branch area pacing has been suggested as an effective alternative to overcome the limitations of His bundle pacing. But, left bundle branch pacing and His bundle pacing have a larger target area and somewhat fewer technological implant challenges. In fact, future studies and large-scale clinical trials are expected to validate HBP and LBBP’s safety, reliability, and long-term performance for physiological pacing in several groups of patients.

## Data Availability

No dataset generated.
